# Conducting International Diploma Course on Leishmaniasis and Its Control in the Islamic Republic of Iran

**Published:** 2019-09-30

**Authors:** Mohammad Reza Yaghoobi-Ershadi, Amir Ahmad Akhavan, Mohammad Reza Shirzadi, Yavar Rassi, Ali Khamesipour, Ahmad Ali Hanafi-Bojd, Hassan Vatandoost

**Affiliations:** 1 Department of Medical Entomology and Vector Control, School of Public Health, Tehran University of Medical Sciences, Tehran, Iran; 2 Zoonosis Control Department, Ministry of Health and Medical Education, Tehran, Iran; 3 Department of Chemical Pollutants and Pesticides, Institute for Environmental Research, Tehran University of Medical Sciences, Tehran, Iran; 4 Center for Research and Training in Skin Diseases and Leprosy (CRTSDL), Tehran University of Medical Sciences, Tehran, Iran

**Keywords:** Leishmaniasis, Diploma course, Disease control, Iran

## Abstract

**Background:**

Leishmaniasis represents the important public health problem in the world. One of the main objectives of World Health organization is capacity building of managers and authorities who are involved with diseases control activities.

**Methods:**

The course was conducted in Esfahan Health Research and Training Center (E.H.R.T.C) in summer 2005 and 2009. The course carried out jointly by the Ministry of Health and Medical Education (MOH) of Iran, World Health Organization-Eastern Mediterranean Regional Office (WHO-EMRO) and School of Public health, Tehran University of Medical Sciences (SPH-TUMS) and designed for medical officers, senior technicians and managers involved in leishmaniasis control. Prior to initiate the course, pre-test evaluations including different subjects on leishmaniasis and its control were carried out. The examinations include multiple choice questions. The duration of the course was 3 weeks. A total of 206 contact hours were taught. The main subjects were Basic epidemiology, Leishmaniasis parasitology, Leishmaniasis entomology, control of vectors and reservoirs, principles of integrated pest management, Field work and Planning. Different methods of teaching including lecture, laboratory, workshop, team work, field exercise and presentation were used. Requirement for achievement of the course was to have at least 60% of the total mark for awarding the diploma certificate.

**Results:**

A total of 45 participants from Iraq, Afghanistan and Iran graduated from this course.

**Conclusion:**

The course is providing the skill for managers, how to combat against disease in their country and is parallel to the policy of the leishmaniasis control for capacity building in endemic areas of their countries.

## Introduction

Human leishmaniasis with a wide clinical spectrum is the neglected form of neglected tropical diseases with a wide variety of parasite species, reservoirs and vectors which are involved in transmission. The causative agent is more than 20 species of the protozoa *Leishmania* and is transmitted to animals and humans through the bite of certain species of female sand flies (1). Out of about 1000 species of sand flies, 93 of them spread leishmaniasis. They are usually 2 millimeter large and can tear the skin in feeding of blood (2).

The disease is extended in 100 countries in the world with a global prevalence of 12 million people and 2 million new cases are reported each year (3, 1). Cases mostly occur in developing countries around the subtropical regions and its incidence is rising significantly. The presence of the disease is linked directly to poverty, but the factors such as social, poor housing, environmental and climate change influence the epidemiology of the disease. More than half of the world's population lives in endemic areas and is at risk of infection (1). Available tools for prevention and control are limited in the world, which means that exposed individuals should take steps to reduce contact with the vector. Furthermore, the health authorities should implement surveillance actions and carry out public health interventions when necessary. Early diagnosis and proper treatment are essential for halting this disease (4).

**Fig. 1.**
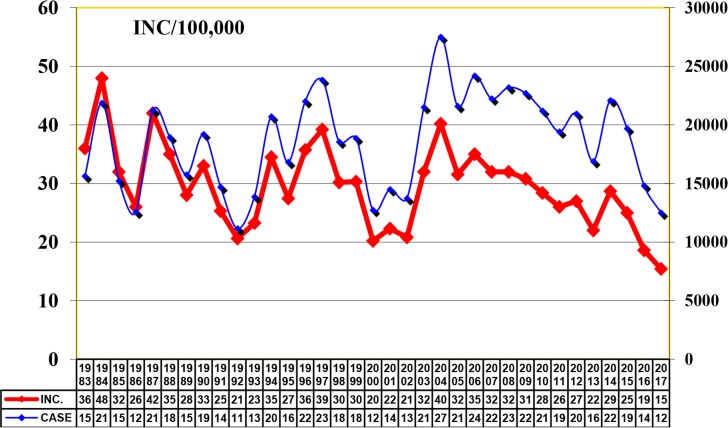
Trend of cutaneous leishmaniasis in Iran, 1983–2017

**Fig. 2.**
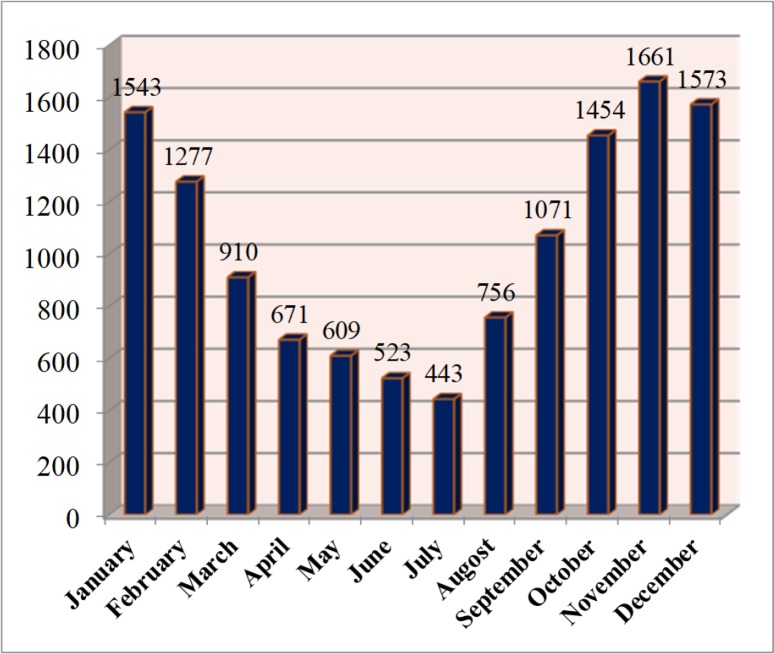
Number of Cutaneous leishmaniasis cases by month, Iran, 2017

**Fig. 3.**
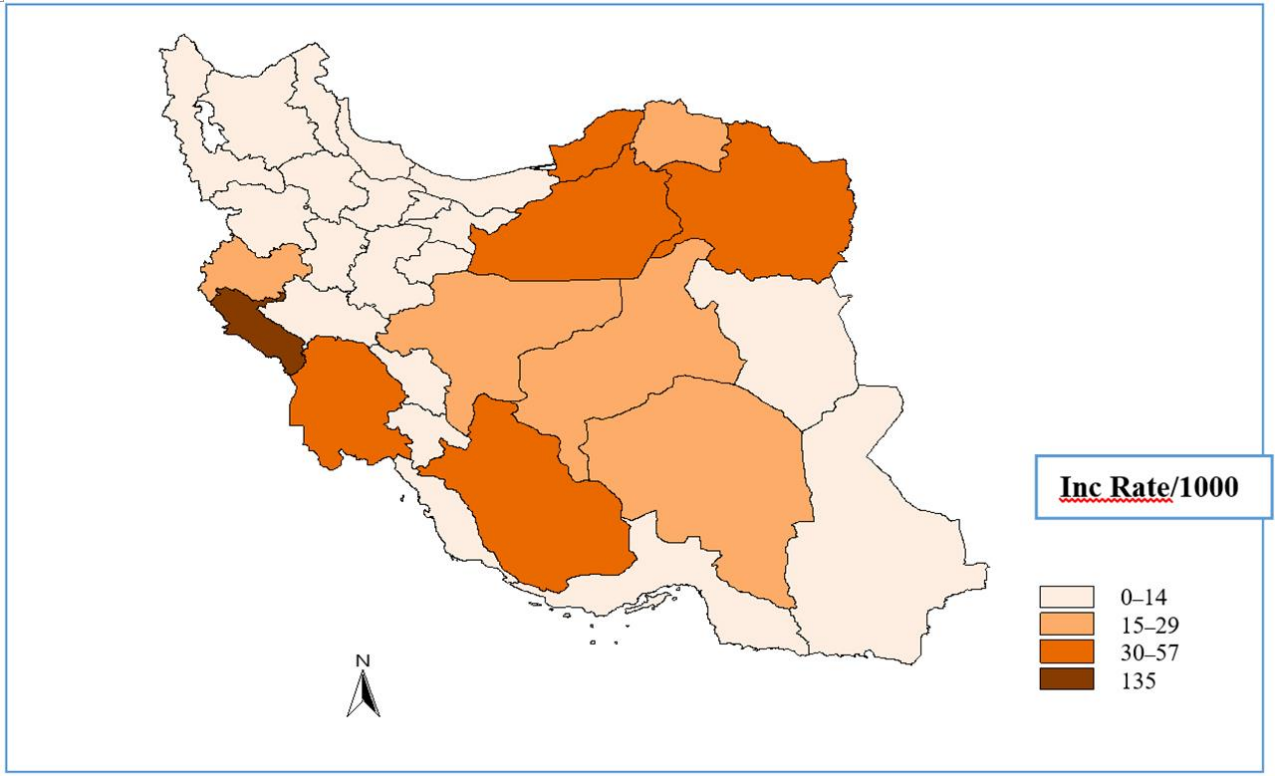
Frequency of cutaneous leishmaniasis cases by province, Iran, 2017

**Fig. 4.**
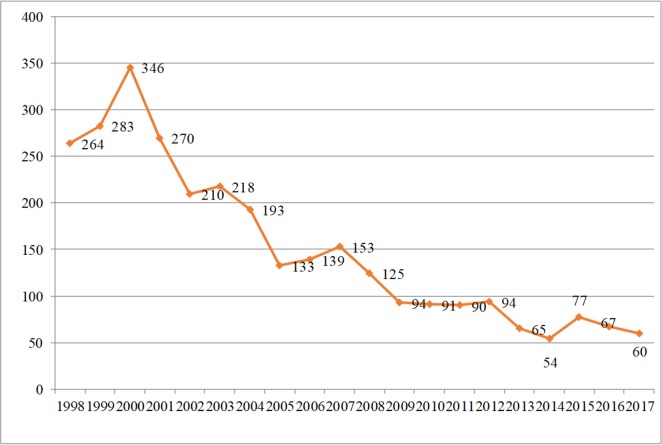
Trend of visceral leishmaniasis in Iran 1998–2017

**Fig. 5.**
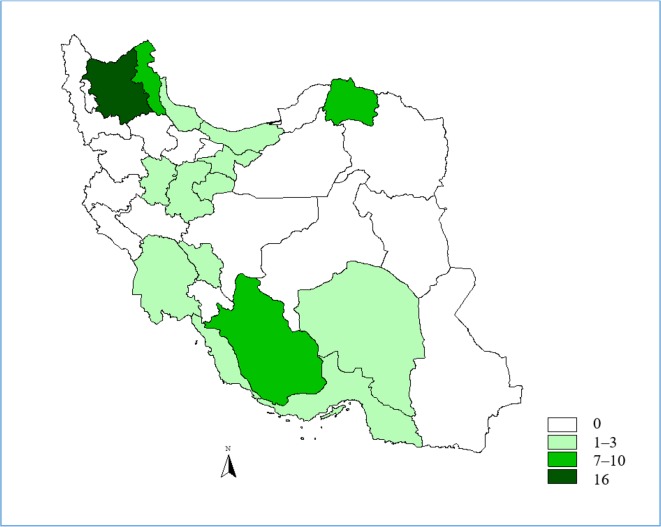
Visceral leishmaniosis cases by province, Iran, 2017

Cutaneous and visceral leishmaniasis are endemic in Iran and continue to be a growing health threat to community development and in the environment. Cutaneous leishmaniasis (CL) occurs in two forms, Anthroponothic Cutaneous Leishmaniasis (ACL) and Zoonotic Cutaneous Leishmaniasis (ZCL) (5). The incidence of CL has been reported between 36–49 cases per 100 000 population during 1983–2017 but in recent years it has had a decreasing trend (Fig. 1). The number of reported CL cases in 2017 was 12491, with minimum of 443 in July and maximum of 1661 in November (Fig. 2). Fig. 3 shows the frequency of cutaneous leishmaniasis cases by province in the country in 2017. The most affected province was Ilam with incidence rate of 135/1000 and the lowest incidence rate (0–11) has been reported from the northwest and southwest areas. A total of 60 cases of VL have been reported from Iran in 2017, the most affected area was Meshkin-Shahr District in the northwest (Fig. 4). The trend of visceral leishmaniasis cases shows a sharp reduction during 1998–2017 (Fig. 5).

The main elements of cutaneous and visceral leishmaniasis control programs in the Islamic Republic of Iran have focused on the establishment of a national committee for leishmaniasis control, are as follows:

Strengthening the leishmaniasis surveillance system and establishing a laboratory network from the national to peripheral level, establishment of standard diagnosis and treatment centers, provision of educational programs for different social levels and revision of the national guideline, standardization of case definition and treatment results, early diagnosis and rapid treatment of patients accompanied by dressing wounds to prevent sand fly bites, reservoir control programmes, collecting the rubble and rubbish tips and vector control programs, applying direct agglutination test in periphery levels for visceral leishmaniasis diagnosis, identification and collection of infected owned and stray dogs in visceral leishmaniasis endemic foci, intersectional coordination with related organizations and institutions (6). Leishmaniasis and its control has been a matter of interest by different researchers at School of Public Health, Tehran University of Medical Sciences (TUMS), in coincidence with the beginning of epidemiological studies on CL in 1965 and have been continued by scientists and young researchers so far. Some of their several publications on different aspects of leishmaniasis is worthy of mention in recent years ( 7–21).

### Course contributors

The course was organized jointly by the Ministry of Health and Medical Education, Islamic Republic of Iran, The School of Public Health and National Institute of Health Research, Tehran University of Medical Sciences, World Health Organization, Eastern Mediterranean Region (EMRO). It provided the participants with the knowledge and skills in different aspects of leishmaniasis and its control, through small group work, field exercises, exchange of experiences and discussions with qualified specialists.

### Contents of the course Epidemiological trends of Leishmaniasis

Description, history and geographical distribution of leishmaniasis in the world and Eastern Mediterranean Regional Office (EMRO), Epidemiology of leishmaniasis in Iran and neighboring countries, effects of climate change and disaster on leishmaniasis situation.

### Treatment

Clinical manifestation and treatment of old world cutaneous and visceral leishmaniasis: physical systematic, interleisonal, oral drugs, management of visceral leishmaniasis

### Immunology of leishmaniasis

Natural immunity and Acquired immunity, Experience of vaccination in leishmaniasis, serological surveillance of asymptomatic visceral leishmaniasis and case detection, leishmaniasis surveillance in Iran.

### Human disease

Methods of studies on human disease: rates, ratios, proportions, prevalence and incidence of leishmaniasis, data presentation: common statistical tests, tables, graphs, charts.

### Leishmaniasis entomology

An overview of vectors and reservoirs of leishmaniasis, biology and ecology of sand flies, Taxonomy of sand flies, identification criteria, explaining of identification keys. Identification of sand fly genera: different sub-genera of *Phlebotomus* genus.

Sampling methods of sand flies: sucking tube, CDC light traps, funnel traps, sticky oil traps and other techniques, preservation, mounting and identification of sand flies, collection of sand flies on human and animal baits, dissection of sand flies for detection and isolation of *Leishmania* parasites, molecular entomology of leishmaniasis vectors.

### Reservoir hosts

Rodent leishmaniasis, biosystematics of rodents, identification of main reservoir hosts, biology, ecology, distribution and behavior of rodents, criteria for incriminating rodents as reservoirs, methods of field and laboratory studies of reservoirs of cutaneous and visceral leishmaniasis, canine visceral leishmaniasis, molecular epizootiology of rodent leishmaniasis.

### Control of leishmaniasis

Different methods of prevention and control of cutaneous and visceral leishmaniasis, methods for evaluation of leishmaniasis control, different methods for elimination of stray dogs, Leishmanization and its effectiveness in the control of CL in Iran, Vaccine for visceral leishmaniasis, principals of integrated pest management (IPM planning), rodenticides and environmental management and sanitation, destroying of chenopods, different methods of rodent control, insecticide susceptibility tests on sand flies, calculation of LT_50_ and LT_90_ using Probit analysis, insecticides recommended by WHO against leishmaniasis vectors, formulation of insecticides for residual spraying, measurement and calculation of insecticides for impregnation of bed nets and curtains, safe use and judicious use of insecticides and precaution measures, insecticide storage (condition of safe storage), space spraying, Ultra Low Volume (ULV), thermal fog.

### Fieldwork and practical demonstrations

Collection of sand flies by different methods from outdoors and indoors in a rural district (field work) around Esfahan, carrying of traps from the field to laboratory, preservation and mounting of collected sand flies, identification of sand fly genera: different sub-genera of *Phlebotomus* genus (laboratory practice), collection of sand flies by different methods (from rodent burrows, on human and animal bait, field work), carrying of traps from the field to laboratory, dissection of collected sand flies for detection and isolation of *Leishmania* parasites, mounting, identification of collected sand flies, collection of rodents by Sherman traps in the field and carrying of them to the laboratory, identification of collected rodent reservoirs, preparations of smears from the ear of each rodent, fixation of slides, staining with Giemsa microscopic examination of slides, isolation of parasites from infected rodents, measurement of rodenticides for application in the field circumstances, preparing of poisoned bait, education of workers, domestic dog examination and control, field application of rodenticides, its monitoring and evaluation impact, visiting households in 3 villages for case finding, examination of people for the presence of ulcers or scars, recording the necessary information on the related forms, preparing smears from ulcers, fixation of slides, staining with Giemsa, microscopically examination of slides, visiting health centers for examination and treatment of patients of CL in Esfahan, different methods of prevention and control of cutaneous and visceral leishmaniasis, methods for evaluation of leishmaniasis control, principals of integrated pest management, Integrated Pets Management (IPM) planning, different methods of rodent control, insecticide susceptibility tests on sand flies, calculation of LT_50_ and LT_90_ using probit analysis, insecticides recommended by WHO against leishmaniasis vectors, formulation of insecticides for residual spraying, measurement and calculation of insecticides for preparation of bed nets and curtains, safe use and judicious use of in insecticides and precaution measures, space spraying, ULV, thermal fog, different parts and function of Hudson pump and thermal fog, measurement and calculation of insecticides for impregnation of bed nets and curtains (practical work), practical work for indoor residual spraying at the station, collection of sand flies by aspirator from the villages, carrying out insecticide susceptibility tests by WHO standard method on collected sand flies, results of susceptibility tests on sand flies: recording and analysis, suggestion of a control program for an Anthroponetic Cutaneous Leishmaniasis (ACL) focus, suggestion of a control program for a Zoonotic Cutaneous Leishmaniasis (ZCL) focus, suggestion of a control program for a Visceral leishmaniasis (VL) focus.

### Evaluation of the course and participants

In evaluating the training course, almost all the training units in the module were covered during the period. There were adequate facilities for the delivery of lectures. These include a well-furnished spacious room for lectures with adequate audio-visual equipment. These include a white board, a slide projector and screen and a dedicated computer for the training. Each participant was provided with a copy of the relevant books and handouts. The participants were given practical field experience and laboratory demonstrations. The laboratory was well equipped with dissecting and compound microscopes which made it possible for each participant to individually and independently do the laboratory work. There was enough reference collection of sandflies available for practical work. All the facilitators tried to make the course as participatory as possible and there was very good relationship between the facilitators and the participants. Almost all the participants through verbal communication rated the entire course as very good, delivery of lectures as excellent and practical demonstrations and fieldwork as good. The onsite accommodation for participants enabled all the activities to be organized on time.

## Conclusions

In general, based on the assessments and interviews, also considering the fact that most of the participants were programme managers and medical health professionals who have had very little knowledge on entomology, vector and reservoir control, the course achieved its purpose of providing them with basic information needed for decision making in disease control activities in their respective countries. The participants and facilitators for the first time shared experiences with regard to vector control activities between different countries. We will recommend that all the participants should be monitored and given the necessary support to enable them contribute to diseases control and elimination activities in their respective countries.
